# Evaluation of Siemens Healthineers’ StrokeSegApp for automated diffusion and perfusion lesion segmentation in patients with ischemic stroke

**DOI:** 10.3389/fneur.2025.1518477

**Published:** 2025-01-24

**Authors:** Lynnet-Samuel J. Teichmann, Ahmed A. Khalil, Kersten Villringer, Jochen B. Fiebach, Stefan Huwer, Eli Gibson, Ivana Galinovic

**Affiliations:** ^1^Center for Stroke Research Berlin, Charité – Universitätsmedizin Berlin, Corporate Member of Freie Universität Berlin and Humboldt-Universität zu Berlin, Berlin, Germany; ^2^Siemens Healthineers AG, Erlangen, Germany

**Keywords:** acute ischemic stroke, automated lesion segmentation, MRI analysis software, diffusion-weighted imaging, perfusion-weighted imaging, Siemens Healthineers StrokeSegApp

## Abstract

**Purpose:**

This study aimed to evaluate the perfomance of Siemens Healthineers’ StrokeSegApp performance in automatically segmenting diffusion and perfusion lesions in patients with acute ischemic stroke and to assess its clinical utility in guiding mechanical thrombectomy decisions.

**Methods:**

This retrospective study used MRI data of acute ischemic stroke patients from the prospective observational single-center 1000Plus study, acquired between September 2008 and June 2013 (clinicaltrials.org; NCT00715533) and manually segmented by radiologists as the ground truth. The performance of the StrokeSegApp was compared against this ground truth using the dice similarity coefficient (DSC) and Bland–Altman plots. The study also evaluated the application’s ability to recommend mechanical thrombectomy based on DEFUSE 2 and 3 trial criteria.

**Results:**

The StrokeSegApp demonstrated a mean DSC of 0.60 (95% CI: 0.57–0.63; *n* = 241) for diffusion deficit segmentation and 0.80 (95% CI: 0.76–0.85; *n* = 56) for perfusion deficit segmentation. The mean volume deviation was 0.49 mL for diffusion lesions and −7.69 mL for perfusion lesions. Out of 56 subjects meeting DEFUSE 2/3 criteria in the cohort, it correctly identified mechanical thrombectomy candidates with a sensitivity of 82.1% (95% CI: 63.1–93.9%) and a specificity of 96.4% (95% CI: 81.7–99.9%).

**Conclusion:**

The Siemens Healthineers’ StrokeSegApp provides accurate automated segmentation of ischemic stroke lesions, comparable to human experts as well as similar commercial software, and shows potential as a reliable tool in clinical decision-making for stroke treatment.

## 1 Introduction

The lifetime prevalence of stroke is estimated at 25% (1). Half of those who survive remain chronically disabled, whereby timely and accurate diagnosis and efficient therapy are crucial for the patient’s outcome (2, 3). Consequently, magnetic resonance imaging (MRI) has become an integral tool in modern stroke diagnostics (4).

As an indispensable sequence, diffusion-weighted imaging (DWI) provides us with information about the areas of the brain likely to be irreversibly damaged by the infarction. The reduction in the diffusivity of water molecules, present in the situation of cytotoxic edema, unmasks the infarct core, which shows up as a drop in the apparent diffusion coefficient (ADC) and an increase in the signal on trace DWI (TraceW) (5).

Perfusion-weighted imaging (PWI), on the other hand, reveals areas of hypoperfused tissue where neuronal dysfunction is potentially reversible. This tissue is potentially at risk of further damage if the insufficient blood flow is not ameliorated. Perfusion maps can be derived from changes in various parameters, such as the relative mean transit time (relMTT), the time to maximum (Tmax), or the cerebral blood flow (CBF) (6).

The juxtaposition of DWI and PWI illustrates the concept of the penumbra, which describes those areas of deficient perfusion that are not part of the irreversibly lost diffusion lesion and thus—despite being currently non-functional—represent vital tissue (7). As these penumbral areas can be saved through timely reperfusion, their quantification has found its way into current recommendations and guidelines for treating stroke patients within the extended or unclear time window (3, 8–10).

Automated image evaluation can support determining penumbra rapidly through image segmentation, whereby deep neural and multilayered convolutional networks in particular have significant potential in neuroradiology (11–14). Although many artificial intelligence (AI) systems already provide reliable results for identifying the ischemic core on MRI (15–19), there are significantly fewer reports on programs that allow for reliable, automatic perfusion segmentation validated on large cohorts (15, 20, 21). In addition, these studies tend to use different statistical approaches, which makes it difficult to compare the applications with each other (22).

The development model of a new generation AI tool from Siemens Healthineers, the research application StrokeSegApp v. 1.3, offers the determination of both lesion types with automatic mismatch calculation. This study had two aims: (a) to provide external and independent validation of the tool’s segmentation results and (b) to shed light on whether the assessment of the penumbra by the program is adequate and would provide clinical benefit in decision-making for acute stroke patients qualifying for mechanical thrombectomy (MT).

## 2 Methods

### 2.1 Study design

This retrospective study used data from the 1000Plus study (clinicaltrials.org; NCT00715533) (23). The protocol received approval from the local Ethics Committee (EA4/026/08), and all study participants gave informed consent for participation in the study. Subjects were eligible for the 1000Plus study if they were admitted with the clinical signs of an acute cerebrovascular event within the last 24 h, were able to undergo an MRI scan, and were at least 19 years old. The MRI protocol included diffusion-weighted imaging (DWI), as well as perfusion imaging (PWI), performed using dynamic susceptibility contrast imaging.

This study is reported following the STARD guidelines.

### 2.2 Participants

**Figure 1:**
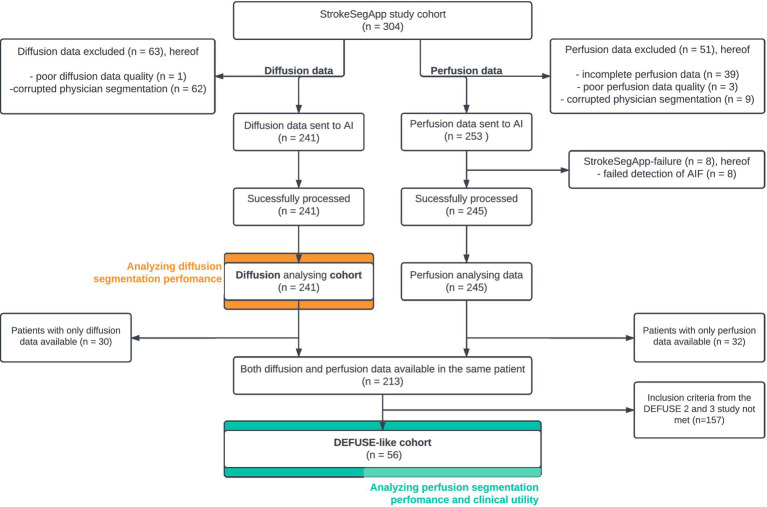
Flowchart detailing the patient inclusion process for data analysis.

Patients were consecutively enrolled between 1 September 2008 and 30 June 2013 at Charité, Campus Benjamin Franklin. In addition to a brain MRI, a detailed medical history was taken. For this study 304 out of 1,437 subjects fulfilled the following inclusion criteria: (a) they had a proven acute stroke, (b) brain perfusion at baseline showed an area of hypoperfusion judged by a radiologist as corresponding to the acute infarct, and (c) this area of hypoperfusion was >10 mL according to the measurements at that time. The last criterion was chosen to only include patients with large enough perfusion deficits to be clinically relevant and credible, i.e., unlikely to be noise or artifacts. As shown in Figure 1, data of diffusion (TraceW and ADC images) and perfusion (dynamic susceptibility contrast perfusion source images) were assessed separately, checked for data quality, and then sent to StrokeSegApp using the software’s implemented batch function. The diffusion data that were successfully evaluated by the program formed the **diffusion cohort** for the subsequent analysis of the accuracy of segmentations conducted by the StrokeSegApp.

To assess perfusion segmentation performance and real-life clinical utility of the StrokeSegApp, an additional subgroup was assembled with patients fulfilling the following criteria: (a) time from symptom onset 0–16 h, (b) NIHSS score > 5 at baseline, and (c) age 18–90 years. All subjects had an ICA or MCA (M1, M2) occlusion on magnetic resonance angiography. These criteria correspond to the inclusion in the DEFUSE 2 and 3 studies (8, 24, 25). These subjects are referred to as the **DEFUSE-like cohort**.

### 2.3 Image acquisition

Our complete imaging protocol has been previously published (22). In short, all examinations were performed on a 3 T MR scanner (MAGNETOM Trio A Tim System, Siemens Healthineers, Forchheim, Germany). DWI was conducted using a spin-echo echo-planar sequence (SE-EPI) [(TE = 93 ms; TR = 7,600 ms; acquisition matrix = 192 × 192 (spatial resolution); slice thickness = 2.5 mm without an interslice gap, b = 0 s/mm^2^ and b = 1,000 s/mm^2^)]. Dynamic susceptibility contrast was conducted using a single-shot gradient-echo EPI sequence [(TE = 29 ms; TR = 1,390 ms; acquisition matrix = 128 × 128 (spatial resolution); slice thickness = 5 mm; interslice gap = 0.5 mm)] with a fixed dosage of 5 mL Gadovist® (Gadobutrol, 1 M, Bayer Schering Pharma AG, Berlin, Germany) followed by 20 mL of saline, both at an injection rate of 5 mL/s.

## 3 Test methods

### 3.1 Ground truth

The ground truth was the manual image delineation, carried out by either of two radiologists (KV or IG), each with more than 5 years of experience in stroke imaging. The radiologists analyzed the images with information on the side and territory of the stroke, but without access to previous MRI images. Diffusion lesions were delineated on TraceW maps with knowledge of the ADC map. Perfusion deficits were delineated in MRIcron on relative mean transit time (relMTT) maps, which were created by the NUM/4 Neuro Perfusion (Siemens Healthineers AG, Forchheim, Germany) using standard singular value deconvolution (sSVD). Diffusion and perfusion lesion volumes were calculated. All these were once again checked for accuracy in 2024 (IG) and, if necessary, re-delineated accordingly.

For the DEFUSE-like cohort, a radiologist with 15 years of experience in stroke imaging (IG) manually segmented the perfusion deficits on the Tmax maps generated by the StrokeSegApp using block circulant singular value deconvolution (oSVD).

### 3.2 Index test

The index test was the AI tool StrokeSegApp version 1.3 (Standalone research package Stroke Segmentation 1.3.0) by Siemens Healthineers AG (Forchheim, Germany), a research application designed to become an integral part of the neuroradiological routine in the MRI-based diagnosis of ischemic strokes and hemorrhagic lesions.

The StrokeSegApp automatically segments acute infarcts in ADC and TraceW images (b-value 1,000 s/mm^2^) as well as the perfusion lesions in perfusion-based dynamic susceptibility contrast images. The application provides both diffusion and perfusion lesion volumes and automatically calculates the PWI-DWI-Mismatch ratio.

Although only the default block circulant singular value deconvolution with oscillation index (oSVD) was used in this study, the program offers three more deconvolutional methods, namely singular value decomposition (sSVD, the original method from Ostergaard (26, 27)), block circulant singular value deconvolution with a fixed cutoff (cSVD) (28), and a Tikhonov regularized Fourier deconvolution (29, 30).

The resulting segmentations can be viewed as overlays on top of the underlying contrast maps (e.g., TraceW and PWI). The perfusion maps generated can also be viewed in the application (relative cerebral blood flow, relative cerebral blood volume, relMTT, and Tmax).

Restricted perfusion lesions (shown as Regions of Interest, ROI) are delineated on Tmax maps. Tmax is calculated using a regularized deconvolution method, namely oSVD (28), with an automatically detected global Arterial Input Function (AIF). The AIF is the average of all AIF candidates from the slice with the most suitable AIF candidates. The AIF candidates are selected from prioritized anatomical regions in the brain, following the areas described by Ebinger et al. and Mouridsen et al. (31, 32).

The lesion segmentation and mirroring of the DWI and PWI lesion are based on deep learning models, which were trained on manually segmented and annotated ground truth data (12,989 for DWI, with a mean age of 70 years, 48% female; 770 for PWI with a mean age of 69 years, 47% female), followed by an internal validation with 1,664 (mean age of 72 years, 52% female) and 98 datasets (mean age of 71, 51% female). Internal testing on 1,897 subjects (mean age of 73 years, 53% female) for detecting infarct on DWI revealed a sensitivity of 0.92 and a specificity of 0.95. For PWI (103 subjects, mean age of 72 years, 52% female), Siemens Healthineers did not determine test accuracy.

The data analyzed in this study were neither used for previous model training nor validation.

The StrokeSegApp reviewed MR images for the analysis without access to original referrals, radiological reports, or neuroradiological diagnoses.

All patient’s datasets were sent to StrokeSegApp thresholding Tmax to 6 s. For index testing, the program’s default setting was used. For two cases with considerable motion, the “PWI Excessive Motion Threshold” was adjusted (15 → 50 mm) to enable successful processing of the images.

## 4 Analysis

### 4.1 Approaches for evaluating metrics of diagnostic precision

#### 4.1.1 Diffusion cohort

To evaluate the diffusion segmentation performance, we first compared the manually segmented lesion volumes with those from the StrokeSegApp. The Bland–Altman plot shows us the volumetric agreement between the ground truth and the index test. We then calculated the Dice–Sørensen coefficient (dice similarity coefficient, DSC) to evaluate the overlap between the two binarized segmentations on the TraceW maps. The DSC is defined as twice the volume of the intersection between two masks divided by the sum of their volumes and serves to quantify their spatial similarity whereby 0 stands for no and 1 for a perfect overlap (22, 33, 34). To assess the impact of lesion size, time to MRI, NIHSS score, and the presence of large vessel occlusion (LVO) on the DSC, a linear model (OLS) was used. Lesion size and time to MRI were included as continuous variables, while LVO was a binary predictor. The NIHSS scores were included as a continuous variable where available, with missing values (*n* = 2) retained in the analysis. The model also included an intercept to account for baseline effects.

#### 4.1.2 DEFUSE-like cohort

For the DEFUSE-like cohort, only patients with both diffusion and perfusion data available were included (see Figure 1). We compared the diffusion data for the new subgroup in the same way as in the diffusion cohort. The evaluation of segmentation performance for perfusion data was carried out by comparing the physician’s Tmax ROI and the StrokeSegApp’s Tmax ROI with thresholding Tmax each to 6 s. Here, too, we created a Bland–Altman plot and calculated the DSC. We also used a linear model in the same way. As all subjects had an LVO as an inclusion criterion, this was not analyzed further here.

Finally, to determine the clinical utility of the software, we assessed the decision for or against mechanical thrombectomy (MT) in line with the criteria of the DEFUSE 3 study, based on the manual and automatically segmented DWI and PWI data. In the DEFUSE 3 study, patients with the following mismatch criteria were eligible for MT: infarct volume < 70 mL, penumbra volume ≥ 15 mL, and mismatch ratio (reduced perfusion/core infarction) ≥1.8 (8). We then calculated sensitivity and specificity for the StrokeSegApp versus the ground truth and furthermore determined the likelihood ratio (likelihood-ratio test, LRT).

The statistical analyses were performed with MATLAB Version 9.1.0.441655 (R2016b),^1^ and statistical graphics were created in IBM SPSS Statistics Version 29.0.0.0 (241).^2^ The MRI images in the figures were visualized using FSLeyes Version 1.4.5.^3^

## 5 Results

### 5.1 Participants

We included 241 subjects for the diffusion segmentation analysis (diffusion cohort) and 56 patients for analyzing perfusion segmentation and indication for MT (DEFUSE-like cohort). Cases of missing sequences or data compromised by excessive patient movement (defined as head motion of more than 50 mm) were excluded. Patients were also rejected if the physician’s original segmentation showed technical processing errors resulting from incorrectly saved physician’s lesion segmentations (ROI) that were not congruent with the original brain map and could not be coregistered. The StrokeSegApp failed to analyze 8 perfusion datasets as it was unable to identify an AIF in these scans (see Figure 1).

**Figure 2:**
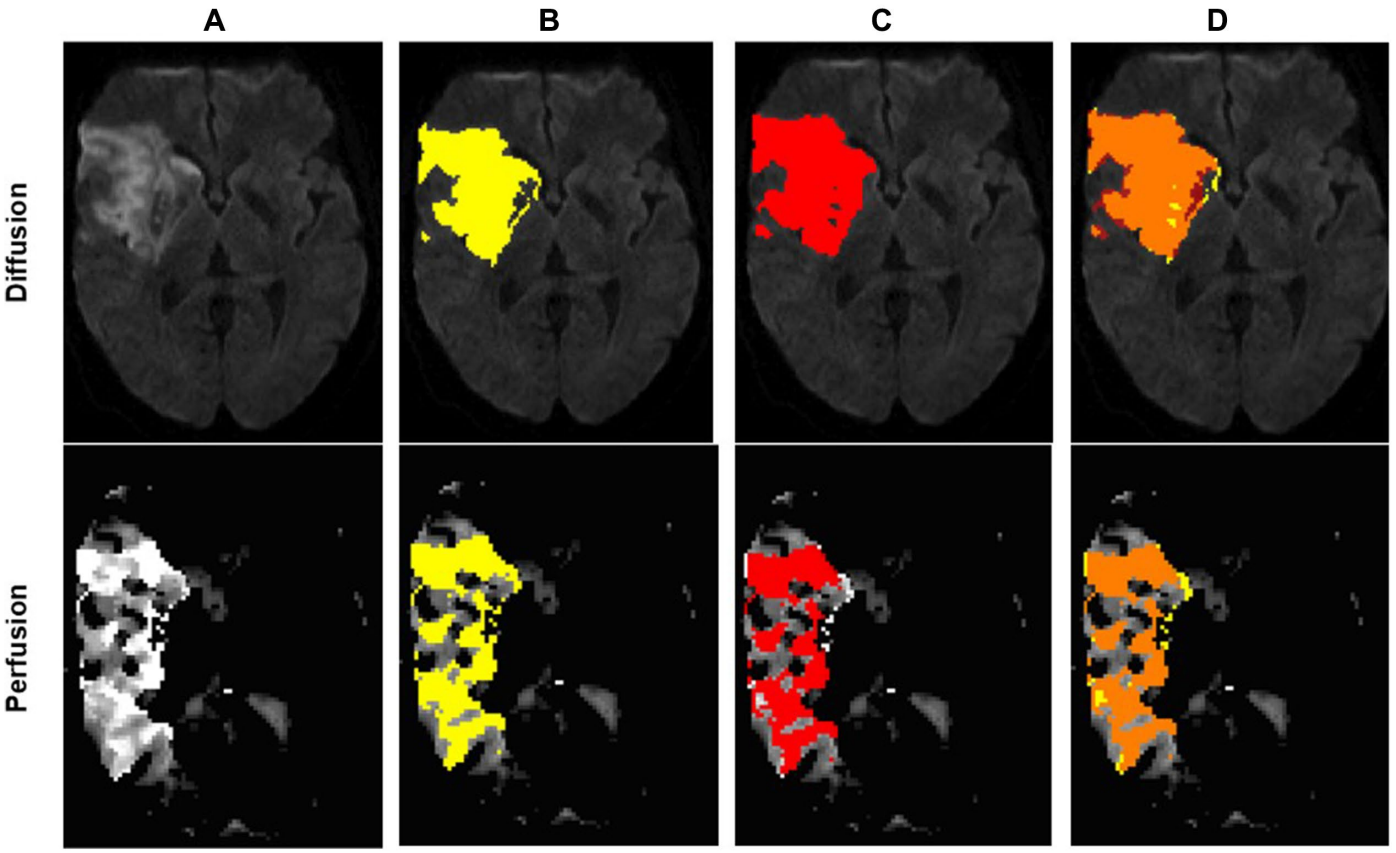
Example of an optimal segmentation of the program compared to the ground truth. The patient had a proximal M1 occlusion on the right side and suffered an infarction of the basal ganglia and the frontal operculum. The diffusion segmentation is shown above on TraceW maps, perfusion can be viewed below on Tmax maps thresholded to 6 s. The diffusion and perfusion segmentation are not displayed in the same slice. DSC for diffusion was 0.87 with an overestimation of the lesion size by the program of 9.63 mL. For perfusion, the dice score was even higher at 0.94, whereby the StrokeSegApp underestimated the deficit slightly by 4.37 mL. Following the DEFUSE 3 criteria, the approaches came to the same conclusion that a mechanical thrombectomy is not indicated because the penumbra is too small. **(A)** Blank scan used for segmentation, **(B)** ground truth ROI, **(C)** StrokeSegApp ROI, and **(D)** both ROI superimposed, whereby orange indicates areas of overlapping mapping, yellow shows areas missed by the StrokeSegApp and red displays diffusion/perfusion deficits overestimated by the application.

Figure 2 shows an example of a successful segmentation of both lesion types in one patient, and Table 1 summarizes the baseline demographic characteristics of participants for both cohorts.

**Table 1:** Baseline patient characteristics.

Variable	Diffusion cohort^1^ (*n* = 241)	DEFUSE-like cohort^1^ (*n* = 56)
Age in years	71 (12)	72 (12)
Sex		
Female	110 (45.6%)	33 (58.9%)
Male	131 (54.4%)	23 (47.1%)
Time window		
Time from stroke to MRI in hours	8.11 (6.9)	5.3 (4.9)
0–6 h	124 (51.5%)	36 (64.3%)
6–16 h	75 (31.1%)	20 (35.7%)
16–24 h	42 (17.4%)	–
LVO distribution		
Cases of LVO	134 (55.6%), hereof	56 (100%), hereof
Only ICA (right, left or both)	23 (17.2%)	8 (14.3%)
Only M1 (right, left or both)	36 (26.9%)	16 (28.6%)
Only M2 (right, left or both)	48 (35.8%)	17 (30.4%)
ICA and MCA (right, left or both)	27 (20.1%)	15 (26.8%)
NIHSS^2^		
0	23 (9.5%)	0 (0%)
1–4	97 (40.2%)	0 (0%)
5–15	97 (40.2%)	36 (64.3%)
16–20	18 (7.5%)	16 (28.6%)
21–42	6 (2.5%)	4 (7.1%)
Diffusion lesion size (mL):		
<0.5	41 (17.0%)	2 (3.6%)
≥0.5 to <10	112 (46.5%)	16 (28.6%)
≥10	88 (36.5%)	38 (67.8%)
Perfusion lesion size (mL):		
<10	–	10 (17.9%)
≥10	–	46 (82.1%)

1 Mean (SD); *n* (%).

2 NIHSS, National Institutes of Health Stroke Scale.

### 5.2 Test results for the diffusion cohort

The StrokeSegApp was able to correctly identify a DWI lesion in 212 out of 241 (88%). Of the 29 missed detections of diffusion lesions, all infarcts were smaller than 10 mL, and 20 (69%) were smaller than 0.5 mL.

**Figure 3:**
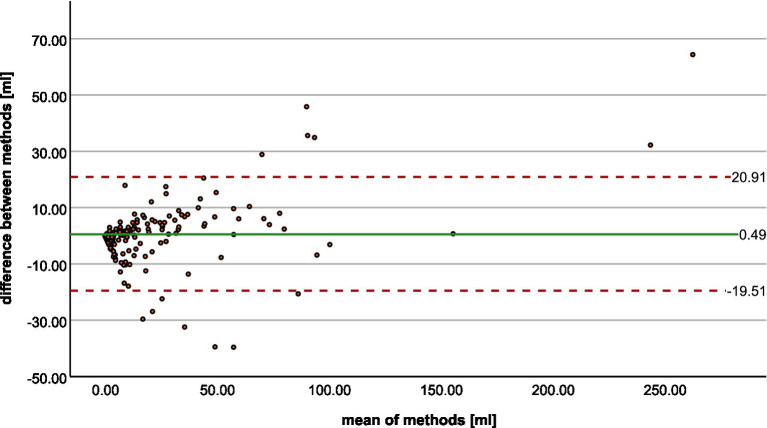
Bland–Altman plot shows the agreement between radiologist and StrokeSegApp for volumes of diffusion lesions.

**Figure 4:**
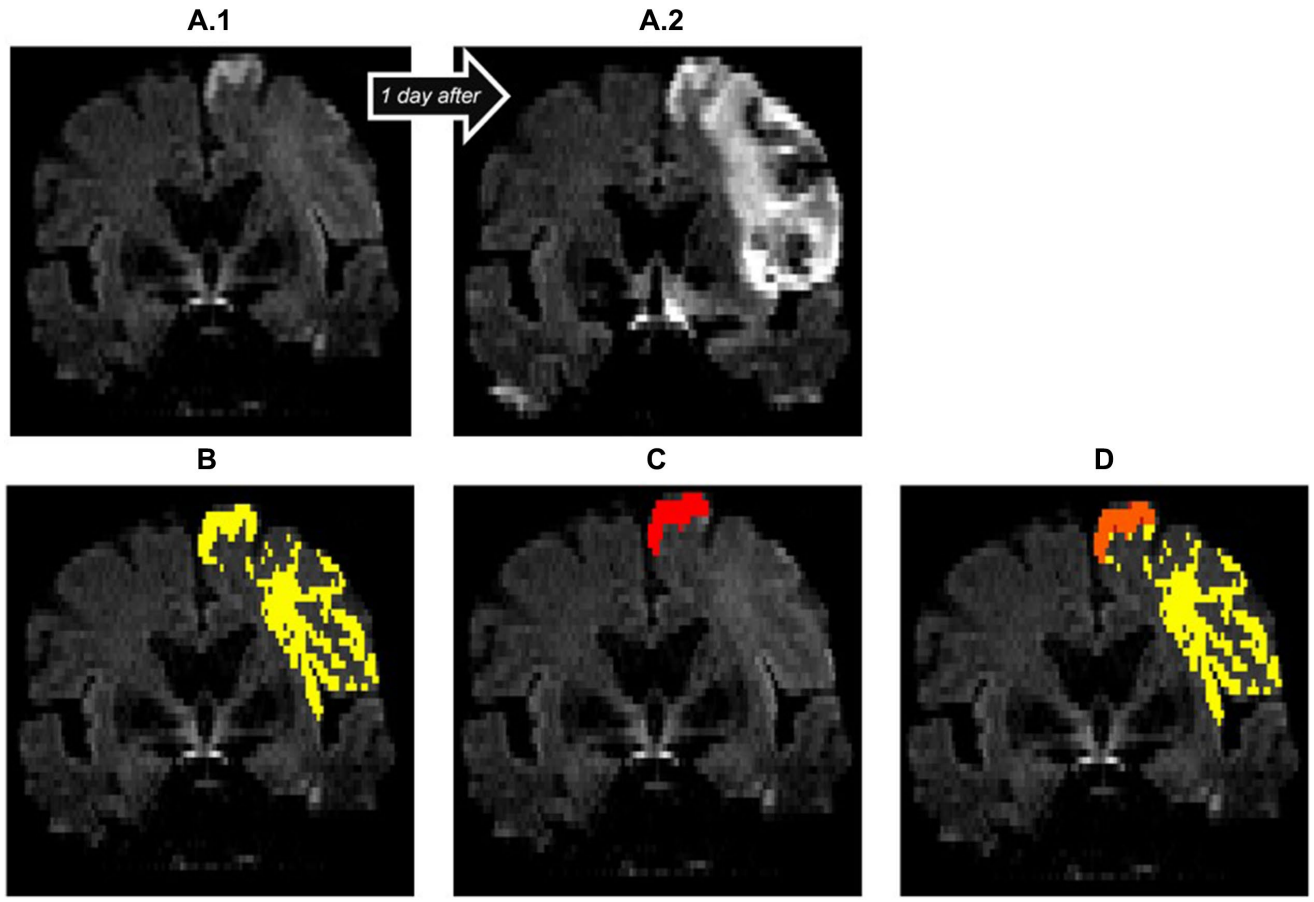
Insufficient diffusion segmentation by the AI on a TraceW map. The clearly hyperintense cortical infarction of the superior frontal gyrus near the cerebral falx was correctly recognized. The remaining faint cortical and subcortical lesions of the frontal lobe, which are hyperintense compared to the contralateral hemisphere on day 1 **(A.1)** and clearly demarcated as part of the acute stroke on follow-up imaging on day 2 **(A.2)**, were not recognized by the StrokeSegApp. The dice score is poor (0.11), the program only delineates 2.13 mL of the 31.73 mL lesion. **(A.1)** blank scan used for segmentation, **(A.2)** follow-up imaging on day 2, **(B)** ground truth ROI, **(C)** StrokeSegApp ROI, **(D)** both ROI superimposed, whereby orange indicates areas of overlapping mapping, yellow shows areas missed by the StrokeSegApp and red displays diffusion deficits overestimated by the application.

**Figure 5:**
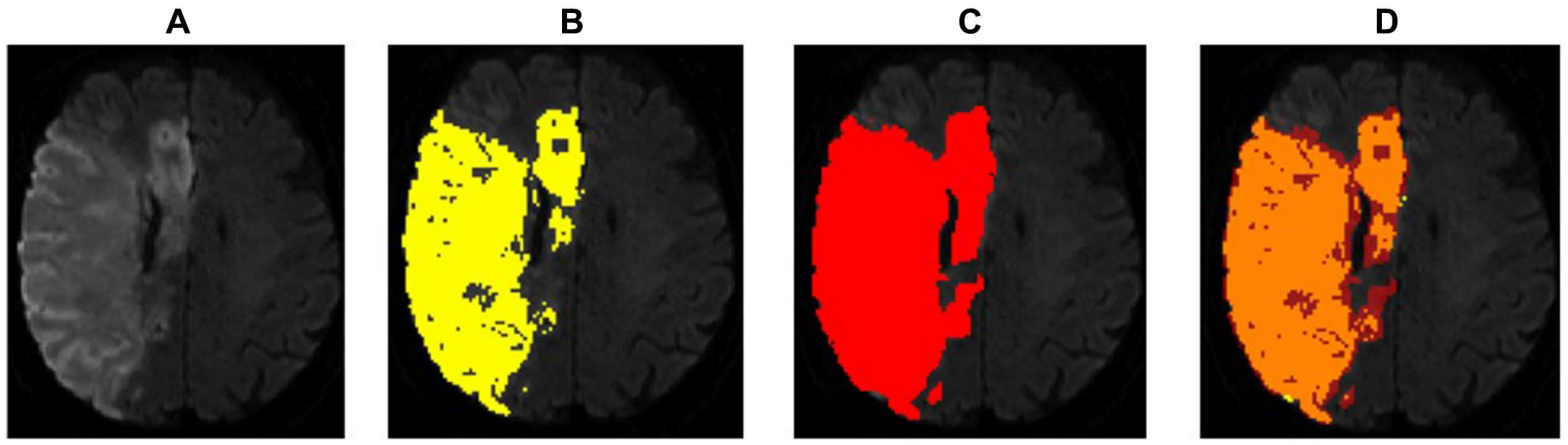
Example of a regionally correct segmentation in DWI with a high dice score (0.87), but a large deviation in lesion volumes. For such large lesions, even slight discrepancies in the thresholds used for delineation by the radiologist and segmentation software, respectively, can lead to large absolute differences in volume. In this case, the StrokeSegApp overestimated the DWI volume by 64.35 mL. However, at that end of the range, such thresholding differences would not typically have clinical relevance; therefore, pure volumetric comparisons are not necessarily the most suitable metric for quantifying a program’s performance. **(A)** Blank scan used for segmentation, **(B)** ground truth ROI, **(C)** StrokeSegApp ROI, and **(D)** both ROI superimposed, whereby orange indicates areas of overlapping mapping, yellow shows areas missed by the StrokeSegApp and red displays diffusion deficits overestimated by the application.

The Bland–Altman plot (Figure 3) shows the volumetric agreement between both methods. The mean difference between the two methods was 0.49 mL, suggesting only a very small systematic difference between the measurements of the two methods. Especially at higher mean values, we see that the datapoints increasingly approach the limits of agreement and extreme outliers occur. Even though a few of these show poor spatial overlap with the ground truth (Figure 4), the majority of cases demonstrate acceptable or good segmentation performance, despite a large volume difference (Figure 5). For lesions <10 mL, the median percentage volume deviation was 22.6% (IQR 7.6–68.9%), while for lesions >10 mL, it was 27.9% (IQR 14.7–53.3%). These findings suggest that, although absolute differences can occasionally be substantial—particularly for larger lesions, which considerably affect the limits of agreement—the relative differences remain consistent and fall within an acceptable range.

**Figure 6:**
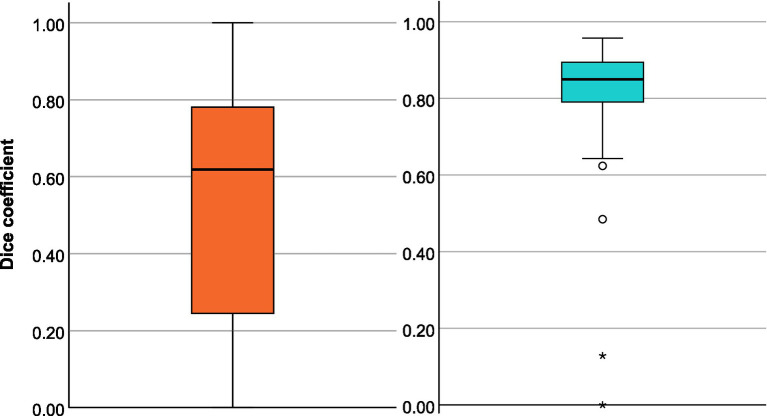
Box plots of dice coefficient (DSC) for the diffusion segmentation **(A)** and the perfusion segmentation **(B)**.

The mean of the DSC for the 212 subjects in whom the software recognized a diffusion lesion was 0.60 (95% CI: 0.57–0.63). The spatial overlap varied greatly depending on the size of the lesion. For infarcts ≥10 mL (*n* = 88), the DSC was 0.72 (95% CI: 0.67–0.76). For small diffusion lesions (<10 mL, *n* = 124), it was markedly lower at 0.53 (95% CI: 0.49–0.57). Figure 6 shows the distribution of the DSC.

To better understand the factors influencing segmentation performance, a linear model was applied. The analysis confirmed that lesion size and time to MRI are significant predictors of the DSC. For every additional 1 mL of lesion size, the DSC increased by 0.003 (95% CI: 0.002–0.005, *p* < 0.001), and for every hour from stroke onset to MRI, the DSC increased by 0.014 (95% CI: 0.009–0.019, *p* < 0.001). However, neither the presence of a large vessel occlusion (*p* = 0.816) nor the NIHSS score (*p* = 0.704) was found to significantly influence DSC values in this dataset.

### 5.3 Test results for DEFUSE-like cohort

We first looked at diffusion and perfusion results separately before analyzing them together as part of the decision for or against MT. Diffusion segmentation performance was similar to that in the diffusion cohort with a DSC of 0.61 (95% CI: 0.53–0.68).

**Figure 7:**
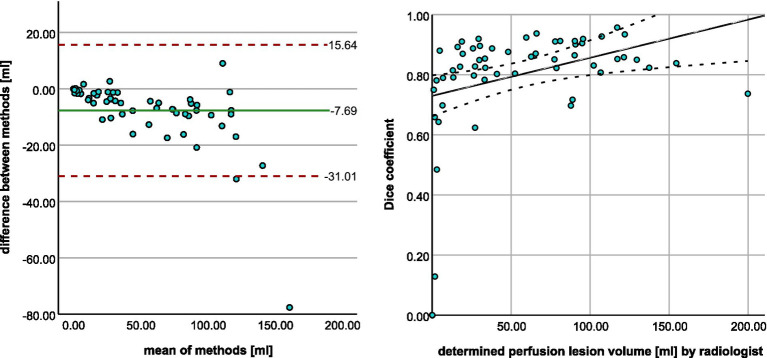
**(A)** Bland–Altman plot shows the agreement between radiologist and StrokeSegApp for volumes of perfusion lesions. **(B)** Scatter plot shows the relationship between manual ROI volume and dice coefficient for the automated segmentation. The line represents smoothed conditional means.

**Figure 8:**
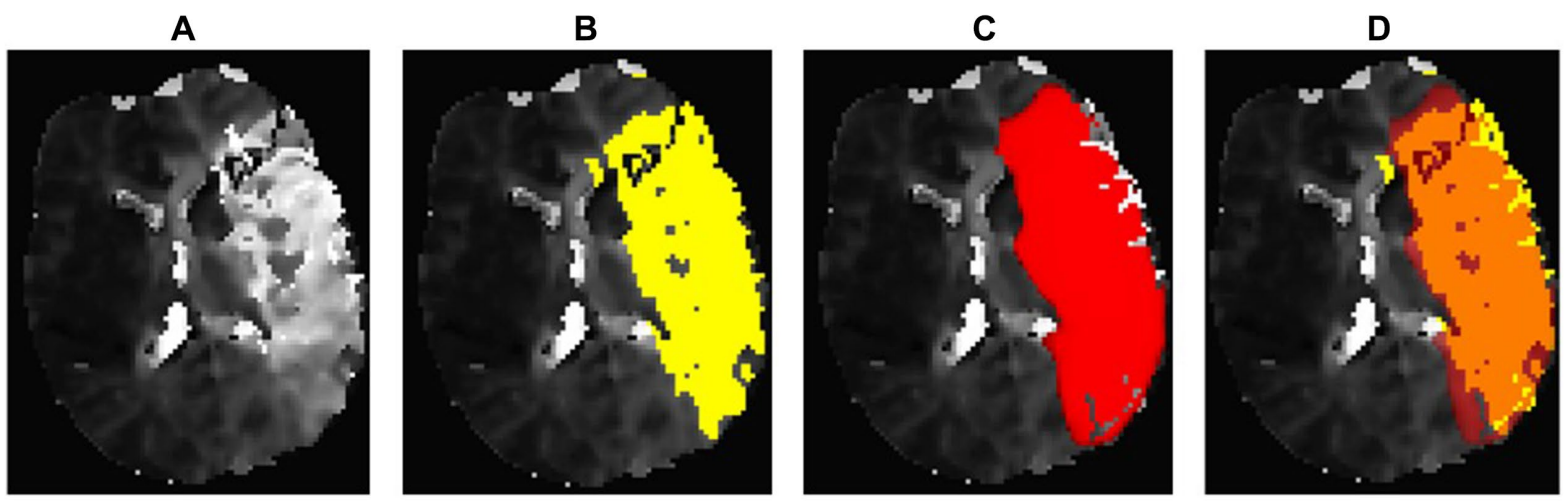
Regionally accurate segmentation in PWI on Tmax maps thresholded to 6 s, achieving a DSC of 0.71. However, it also displays a significant discrepancy in lesion volumes. The StrokeSegApp underestimates the extensive deficit, which measures 199.83 mL, by 77.65 mL. This case is representative of the fact that a comparison of volumes can only be used to a limited extent to assess segmentation performance. **(A)** Blank scan used for segmentation, **(B)** ground truth ROI, **(C)** StrokeSegApp ROI, and **(D)** both ROI superimposed, whereby orange indicates areas of overlapping mapping, yellow shows areas missed by the StrokeSegApp, and red displays perfusion deficits overestimated by the application.

In terms of perfusion, the program was able to correctly determine the presence of hypoperfusion corresponding to the area of the acute stroke in all 56 cases. Volumetric agreement on perfusion lesion volumes is shown in Figure 7A. With a mean deviation of −7.69 mL, the StrokeSegApp consistently and slightly underestimates the size of the perfusion lesions. As the size of the deficit grows, we can see that the scatter of the datapoints increases volume-wise. Nevertheless, large volume deviations—and even outliers—are usually accompanied by good segmentation performance in terms of DSC (see Figure 8). Large lesions are associated with better automated segmentation performance than small ones as seen in Figure 7B.

The mean DSC for perfusion lesions was 0.80 (95% CI 0.76–0.85). Focused on the lesions that have a volume of ≥10 mL (*n* = 46), we see a DSC of 0.85 (95% CI: 0.83–0.87). See Figure 6 for a summary of the data’s distribution. The OLS explained 20.2% of the variability in DSC (*R*^2^ = 0.202, *p* = 0.0079). Lesion size (*p* = 0.093), and time to MRI (*p* = 0.082) showed trends toward significance, with a DSC increase of 0.0009 per 1 mL lesion size and 0.0077 per hour from stroke onset to MRI, respectively. The NIHSS (*p* = 0.222) did not significantly influence DSC.

To evaluate the clinical utility of the StrokeSegApp, we compared the decision for and against MT between the physician and the software. Out of 28 cases where MT would have been indicated based on the ground truth, StrokeSegApp recommended MT for 23 subjects (sensitivity of 82.1%; 95% CI: 63.1–93.9%). In cases where MT would not be recommended by the ground truth (*n* = 28), the AI segmentation reached the same conclusion in 27 patients (specificity of 96.4%; 95% CI: 81.7–99.9%); see Table 2 for cross-tabulation. In all five cases where the values determined by the program did not indicate MT, this was because the automatically calculated penumbra was too small. In one case, the diffusion lesion was overestimated. Visual inspection confirmed that the areas identified by the StrokeSegApp aligned well with the infarct core and the corresponding perfusion deficit. However, the tool’s delineation boundaries differed enough from those of the radiologist to result in a significant volumetric discrepancy. The likelihood ratios LR+ and LR− were 22.81 and 0.19. The large positive LR (>10) implies that a positive test result greatly increases the probability of recommending MT. Conversely, an LR− of 0.19 suggests that a negative test result is moderately effective in ruling out the need for MT.

**Table 2:** Cross-tabulation of decisions for or against MT based on data from the radiologist (ground truth) and the StrokeSegApp.

	Ground truth: MT indicated	Ground truth: MT not indicated	Total
StrokeSegApp: MT indicated	23	1	24
StrokeSegApp: MT not indicated	5	27	32
Total	28	28	56

Sensitivity	Specificity	Likelihood Ratio (LR)	
		LR+	LR−
82.1%	96.4%	22.81	0.19
95% CI: 63.1–93.9%	95% CI: 81.7–99.9%	95% CI: 5.13–631	95% CI: 0.06–0.45

## 6 Discussion

We carried out an independent evaluation of the StrokeSegApp using a large retrospective real-life cohort of patients (>200 datasets) who underwent MRI screening for acute ischemic stroke. The StrokeSegApp showed very good reliability and accuracy in recognizing acute ischemic changes on DWI and was also able to segment the ischemic core with a mean difference of 0.49 mL compared to human radiology experts. In a fully automated fashion, the AI was able to produce usable perfusion maps for 97% of patients in the sample. Its segmentations of hypoperfusion showed a high degree of spatial overlap with a human rater and a mean volumetric difference of only 7.68 mL. The linear model results confirm that lesion size and time to MRI are the primary determinants of segmentation performance, with larger lesions and later imaging times associated with higher dice similarity coefficient values. For the diffusion cohort, these effects were pronounced, with lesion size and timing significantly influencing DSC. In the DEFUSE-like cohort, lesion size and time to MRI showed trends toward significance, but their impact was less marked, likely reflecting the homogeneity of patients with large vessel occlusion. The NIHSS did not significantly influence DSC in either cohort, suggesting that lesion size and time to MRI already account for stroke severity and occlusion-related variability. These findings align with expectations and emphasize the importance of lesion size and timing in determining segmentation accuracy. The lower DSC observed in smaller lesions should be understood in the context that segmentation errors in objects with a small volume are penalized more harshly. Given their clinical importance in early-stage stroke assessment, proven strategies to improve the segmentation of small lesions could include, for example, data enrichment with additional real or synthetic cases (35) and lesion-volume-based data sampling and loss functions (36).

When the StrokeSegApp is used as part of a decision matrix for recanalization in patients with a large vessel occlusion in the anterior circulation (based on the criteria of the DEFUSE 2 and DEFUSE 3 clinical trials), it would provide 82% sensitivity and 96% specificity in selecting candidates for reperfusion therapy, compared to a human gold standard. While the sensitivity result is promising, the automated analysis would have ruled out thrombectomy in an additional five cases. Even though visual inspection confirmed that the areas identified by the software corresponded well to the infarct core and matching perfusion deficit, the delineation boundaries differed sufficiently from those selected by the radiologist in all cases, leading to rejection due to a PWI/DWI mismatch <1.8. Such strict decision criteria are necessary in large clinical trials such as DEFUSE, but they hold less significance in routine clinical practice, where information is interpreted more holistically without adhering rigidly to specific thresholds. Additionally, some degree of interrater variability is expected, both in human and software analyses, which underscores the challenges of achieving perfect alignment.

A direct comparison of Siemens Healthineers’ software with other programs that are commercially available cannot be made, as publications on segmentation performance in ischemic strokes rarely include a comparison of spatial overlap and volumetric congruence between the software providing automated segmentation and a ground truth. In contrast, the number of publications on the performance of non-commercial applications for segmenting diffusion lesions is quite high—mostly with the provision of dice scores. In a study also using data from the 1000Plus study (but not using the same cohort as here), the algorithm developed by the research group achieved a DSC of 0.43 compared to manual segmentations (37). The ISLES challenges—an open competition to foster the development and innovation of advanced tools for ischemic stroke analysis—reflect the development in ischemic stroke lesion segmentation of non-profit participants as a steady improvement. While the top 2018 model reached a dice score of 0.51, recent advancements have pushed this to 0.69, and the 2022 challenge has seen scores as high as 0.78, demonstrating progress in segmentation accuracy across different imaging modalities (38).

The StrokeSegApp can compete with those results and performs comparably to commercial software solutions on which data are available. In one publication (*n* = 131), RapidAI showed a mean deviation of diffusion lesion volumes of −1.95 mL (StrokeSegApp: 0.49 mL) with limits of agreement of −12.89 mL to 9 mL (StrokeSegApp: −19.51 mL to 20.91 mL) (39). The e-Stroke software distributed by Brainomix showed a mean volume deviation of 4 mL in the segmentation of CT perfusion for determining the infarct core (*n* = 551) (40). OleaSphere from Olea Medical achieved a dice score of 0.52 (StrokeSegApp: 0.60) compared to a ground truth segmented by physicians using DWI (*n* = 75) (41).

Regarding perfusion, a study using RapidAI reported a mean deviation of 7.60 mL (StrokeSegApp: −7.69 mL) from a reference determined by radiologists (*n* = 18) (25). In using more than just a mere volumetric comparison, our voxel-based approach to the validation of perfusion segmentation against a ground truth adds an additional level of rigor in terms of validation of automated segmentation software as compared to the current literature. This underlines the need for a standardized guideline for evaluation metrics, as proposed by Müller et al. (22), which helps manufacturers align their evaluations and enables a robust comparison of the relative strengths and weaknesses of different AI applications. With a dice score of 0.8 (or 0.85 for lesions ≥10 mL), the StrokeSegApp shows very good overlap with human raters and sets standards for current and future applications.

In a real-time clinical context, the StrokeSegApp could be integrated either directly at the scanner or at a connected processing node, where the calculation is automatically triggered upon the arrival of new data. With both approaches the resulting DICOM image and report data could be delivered to the PACS reading station. Current calculation times for the entire stroke segmentation pipeline range from approximately 1–2 min on standard computer hardware making the use of the software feasible in an actual clinical workflow. The processing time can increase considerably in cases with substantial artifacts, such as excessive patient movement. Beyond delays, poor contrast and strong noise can also adversely affect the analysis. This occurs in two main ways. First, poor contrast in the original perfusion-weighted images, particularly when the contrast bolus dip is reduced, can degrade the quality of pre-fusion map generation. Second, segmentation performance may decline, as it is closely tied to the accuracy of the perfusion maps. To address these challenges, Siemens has implemented a method in the current software version that automatically rejects processing if minimal quality requirements are not met. The rejection criteria include: (a) insufficient contrast bolus drop in the PWI data relative to baseline noise, (b) excessive motion artifacts between timepoints, and (c) an arterial input function that is too weak to produce accurate perfusion maps. The output includes, in addition to the endpoint results (mismatch volumes and ratio) visualizations of segmented lesions in the context of the original data, details about the arterial input function used in the calculation, and brain extraction masks. This additional information aids radiologists in decision-making, builds trust in the derived values, and helps identify errors in cases where the automatic calculation fails. Incorporating mechanisms for users to provide feedback about the application to the manufacturer would further enhance its usability and reliability.

The integration of AI into neuroimaging, particularly in the context of segmenting stroke lesions, demonstrates the transformative potential for diagnostic and therapeutic advancements but also raises significant ethical concerns. A core challenge lies in the dependency of AI models on the quality and consistency of input data, spanning training, validation, and clinical application phases. Errors stemming from algorithmic biases in segmentation (42) can directly affect clinical decisions and patient outcomes. Moreover, ensuring a diverse and representative dataset for training and validation is critical to addressing disparities in healthcare (43), especially for marginalized groups, who risk underrepresentation in neuroimaging repositories (44). The necessity for transparency in training strategies, algorithmic decisions, and the management of sensitive patient data is paramount (44). This transparency ensures ethical AI deployment, builds trust, and enhances clinical applicability. Collaborative interdisciplinary approaches that merge clinical, technical, and ethical expertise are essential to mitigate biases and promote equitable AI applications across diverse populations.

Our results should be viewed in the context of the study’s limitations. This is a single-center retrospective analysis of data acquired between 2009 and 2013; therefore, we cannot rule out that the StrokeSegApp may perform differently on data acquired using contemporary scanning protocols. The ground truth comes from manual segmentation, which is prone to subjective variability.

## 7 Conclusion

The Siemens Healthineers StrokeSegApp provides automated segmentation of diffusion and dynamic susceptibility contrast perfusion on MR images in acute ischemic stroke patients, achieving a high degree of accuracy compared to a human expert. Therefore, it is comparable to other existing and already commercially available solutions.

## Data Availability

The raw data supporting the conclusions of this article will be made available by the authors without undue reservation.

## Footnotes

^1^MathWorks, Natick, MA, USA, https://www.mathworks.com.

^2^IBM Corp., Armonk, NY, USA, https://www.ibm.com/products/spss-statistics.

^3^FMRIB Analysis Group, University of Oxford, Oxford, UK, https://fsl.fmrib.ox.ac.uk/fsl/fsleyes.
